# Temporal trends in viral setpoint and peak CD4^+^ cell count soon after HIV-1 seroconversion

**DOI:** 10.1097/QAD.0000000000004527

**Published:** 2026-04-22

**Authors:** Nikos Pantazis, Caroline Sabin, Dominique Costagliola, Sophie Grabar, Christina Carlander, John Gill, Ard van Sighem, Inma Jarrin, Laurence Meyer, Elisa Ruiz-Burga, Shema Tariq, Bruno Spire, Fiona Burns, Giota Touloumi

**Affiliations:** a Medical School, National and Kapodistrian University of Athens, Athens, Greece; b Institute for Global Health, University College London, UK; c Sorbonne Université, INSERM, Institut Pierre Louis d’Epidémiologie et de Santé Publique, AP-HP, Hôpital St Antoine, Paris, France; d Department of Infectious Diseases, Karolinska University Hospital, Stockholm, Sweden; e Southern Alberta HIV Clinic, Calgary, Alberta, Canada; f Stichting hiv monitoring, Amsterdam, The Netherlands; g National Centre of Epidemiology, Carlos III Health Institute, Madrid, Spain; h INSERM CESP U1018, APHP Hôpital de Bicêtre, Le Kremlin-Bicêtre, Paris-Saclay University, Gif-sur-Yvette, France; i Institute of Health Informatics, University College London, UK; j Inserm, IRD, SESSTIM, ISSPAM, Aix-Marseille Université, Marseille, France.

**Keywords:** CD4, HIV-1 RNA, seroconversion, setpoint viral load, virulence

## Abstract

**Objectives:**

To examine whether HIV-1 virulence, as measured by prognostic markers soon after seroconversion, has changed over time and across subgroups in the context of widespread ART availability.

**Design:**

Observational cohort study of individuals with well estimated HIV-1 seroconversion dates recruited from clinics in eight European countries and Canada (CASCADE Collaboration).

**Methods:**

We analyzed data from 21 933 participants who seroconverted 1980–2014, while ART-naïve and AIDS-free. Piecewise linear mixed models with one knot at 1 year after seroconversion for log_10_ HIV-1 RNA (setpoint viral load, SPVL) and 4 months for square-root CD4^+^ cell count (peak CD4) were fitted. Models adjusted for age, sex/probable exposure route, region of origin, diagnosis during acute infection, and assay type.

**Results:**

SPVL increased and peak CD4 declined over time until approximately 2000, then stabilized, with a mild reversal after 2005. Estimated (95% CI) average SPVL for those seroconverting on 1/1/1980, 1/1/1995, 1/1/2005, and 31/12/2014 was 3.2 (3.0–3.4), 4.0 (3.9–4.1), 4.3 (4.2–4.4), and 4.2 (4.1–4.3) log10 copies/ml, respectively. Corresponding estimates for peak CD4 were 960 (898–1025), 615 (584–648), 521 (492–551), and 560 (526–594) cells/μl. The increase in SPVL up to 2005 was less pronounced in European/North American MSM (1.0 log_10_ copies/ml) than in men and women with heterosexual exposure from the same region or from Africa (1.5 and 1.4 log_10_ copies/ml, respectively).

**Conclusions:**

SPVL and peak CD4 stabilized by 2000–2005, consistent with HIV-1 adaptation to the population. The subsequent trend reversal, most evident among MSM, warrants ongoing monitoring.

## Introduction

Like other RNA viruses, HIV-1 is highly error-prone during replication, resulting in a high mutation rate that enables adaptation to its human host [1]. This virus-host relationship is thought to follow a virulence-transmission trade-off model, balancing viral survival through disease induction without killing the host [2,3].

Due to the impact of potent antiretroviral therapy (ART) on immune function [4,5] and life expectancy [6], fewer people with HIV now experience an AIDS-defining event. Thus, time from infection to AIDS or death is no longer a practical measure of HIV virulence. Instead, two routinely measured laboratory markers: plasma HIV-1 RNA, from which the setpoint viral load (SPVL) can be estimated, and CD4^+^ cell count, are commonly used as proxies of HIV-1 virulence. SPVL reflects the balance between viral replication and host immune control and is associated with both disease progression and transmissibility, whereas CD4^+^ cell count reflects immune depletion and the pace of disease progression. These markers, measured during untreated infection and preferably early in the course of disease, reflect early infection dynamics before treatment or advanced progression [2,7].

In 2014, we reported on trends in these markers using CASCADE data from 15 875 individuals with well estimated HIV-1 seroconversion dates, observing increased virulence and transmissibility over 1979–2008, with a possible plateau after 2002 [8].

In 2025, we aimed to examine whether trends in these markers had changed since 2008, given the wider global ART access and, if so, whether changes were similar across age groups, sex at birth and probable route of HIV-1 exposure, geographic region of origin and diagnosis during acute infection.

This is important because the development of control strategies for HIV-1 is dependent in part on understanding how the virus has evolved in humans and the implications for the development of prophylactic and therapeutic vaccines [9]. Furthermore, the rapid evolution of HIV-1 increases the risk of mutations [10] which encode for drug resistance, with clear relevance to the continued success of treatment.

## Methods

We pooled retrospective clinical data from cohorts participating in the CASCADE Collaboration and additional cohorts from a prior publication [8]. This enabled inclusion of longitudinal data from the early HIV-1 epidemic in Europe and North America.

CASCADE is a multinational mixed methods study of HIV-1 cohorts of individuals with well estimated dates of HIV-1 seroconversion recruiting from clinics in France, Netherlands, Spain, Sweden, Greece, Canada, and the UK [11]. All participants were ≥16 years at HIV-1 diagnosis and fulfilled at least one of the following criteria: a positive HIV-1 antibody test within 12 months of a negative HIV-1 antibody test, laboratory evidence of seroconversion (HIV-1 antibody negative with positive RT-PCR, an “incident” test using a recent incident testing algorithm assay (RITA), an equivocal HIV-1 antibody test supported by a repeat test within a 2-week period showing a rising optical density, or p24 antigen positivity supported by neutralization or PCR or clinical manifestations of symptomatic HIV seroconversion illness supported by antigen positivity and <4 bands positive on Western Blot). Seroconversion dates were estimated as the midpoint between the last negative and first positive HIV-1 antibody tests, as the date of sample collection for other laboratory methods, or the date of onset of seroconversion illness, if recorded. Participants included from our previous publication [8] were recruited from clinics in Spain, Italy, Germany and the UK and met the same eligibility criteria.

For this analysis, individuals also needed ≥1 pre-ART or pre-AIDS HIV-1 RNA viral load measurement (VL) and CD4^+^ cell count (CD4), and a seroconversion date between January 1, 1980 and December 31, 2014. We excluded measurements taken during ART or after AIDS diagnosis, as these alter the natural course of the markers. Individuals seroconverting after January 1, 2015 were excluded, as early ART initiation precludes reliable estimation of peak CD4 and viral setpoint. For all participants, CD4 and viral load measurements were obtained after the first confirmed HIV-positive test. Consequently, our data start from the early postseroconversion recovery phase rather than the brief period immediately following infection thus short-term CD4 and viral load fluctuations that occur soon after infection are not represented in our analyses.

### Ethics approval

Ethics approval for data pooling was obtained by cohort leads with informed signed consent. Approving committees are listed in the Appendix, Supplemental Digital Content.

### Statistical analysis

Demographic and clinical characteristics are summarized using standard measures [median/interquartile range (IQR) for continuous variables; absolute and relative frequencies for categorical variables].

Initial exploration of VL (log_10_ transformed) and CD4 (square-root transformed) evolution during HIV-1 natural history (i.e. before ART initiation or AIDS diagnosis) was performed using 3-level linear mixed models (marker measurements nested within individuals nested within cohorts). Time since seroconversion was entered in the fixed part of these models through restricted cubic splines with 7 knots at the 2.5, 18.33, 34.17, 50, 65.83, 81.67, and 97.5 percentile of the distribution of the time of the last measurement per individual [12]. Models included a random intercept at the cohort level and a random intercept along with a spline function of time since seroconversion with 3 knots at the 5, 10, and 90 percentiles of the distribution [12] of the time of the last measurement per individual with an unstructured covariance matrix at the individual level.

For the main analyses, the fixed part of the models was simplified using a piecewise linear function with one knot at 1 year after seroconversion for viral load (VL) and at 4 months for CD4 guided by examination of the data. We refer to these values as SPVL and peak CD4 hereinafter, respectively.

The main covariate of interest was the calendar time of seroconversion which was allowed to affect SPVL and peak CD4 and to also interact with the two linear time terms through a restricted cubic spline with 5 knots at the 5, 27.5, 50, 72.5, and 95 percentile [12]. The main models included adjustments for age at seroconversion, sex at birth and probable route of exposure combined, region of origin and diagnosis during acute infection (defined as a <30 days HIV test interval, seroconversion illness or diagnosis through other laboratory evidence of seroconversion), and VL quantification assay (only for SPVL model). Additional analyses were based on: a) models with additional interactions to investigate whether the effects of calendar time of seroconversion differed by age, sex at birth/probable route of exposure combinations, and region of origin, and b) unadjusted models fitted to subgroups of women having sex with men, men having sex with men (MSM), men who do not report sex with men, and individuals originating from Africa.

## Results

Of the 35 729 individuals in the combined CASCADE database, 29 411 (82.3%) seroconverted January 1, 1980 to December 31, 2014. Of these, 21 933 (74.6%) had at least one VL and one CD4 measurement taken while ART naïve and AIDS-free. Their demographic and clinical characteristics are summarized in Table 1. Median (IQR) age at estimated seroconversion was 32 years (26, 40), the majority were MSM (68.9%), and of European or North American origin (82.6%). The median (IQR) number of VL and CD4 measurements was 3 (1, 7) and 4 (2, 8), respectively, and the last VL and CD4 measurements were taken 19 (6, 50) and 20 (6, 51) months after seroconversion.

**Table 1:** Demographic and clinical characteristics and marker measurements by year of seroconversion (SC).

	Calendar time of HIV-1 seroconversion				
Variable	1980–1995	1996–2004	2005–2009	2010–2014	Overall
	*n* *=* *2860 (13.0%)*	*n* *=* *6491 (29.6%)*	*n* *=* *6136 (28.0%)*	*n* *=* *6446 (29.4%)*	*N* *=* *21933 (100%)*
Age at estimated SC (years)	27 (23, 33)	32 (27, 39)	34 (28, 41)	33 (27, 42)	32 (26, 40)
Sex at birth and probable exposure					
*– Men having sex with men*	1362 (47.6%)	3899 (60.1%)	4653 (75.8%)	5194 (80.6%)	15108 (68.9%)
*– People who inject drugs*	606 (21.2%)	384 (5.9%)	92 (1.5%)	72 (1.1%)	1154 (5.3%)
*– Men having sex with women*	265 (9.3%)	771 (11.9%)	493 (8.0%)	446 (6.9%)	1975 (9.0%)
*– Women having sex with men*	534 (18.7%)	1157 (17.8%)	701 (11.4%)	517 (8.0%)	2909 (13.3%)
*– Other-Not known*	93 (3.3%)	280 (4.3%)	197 (3.2%)	217 (3.4%)	787 (3.6%)
Region of origin						
*– Europe/N. America*	2542 (88.9%)	5353 (82.5%)	5052 (82.3%)	5167 (80.2%)	18114 (82.6%)
*– Africa*	95 (3.3%)	585 (9.0%)	470 (7.7%)	417 (6.5%)	1567 (7.1%)
*– Lat. America/Caribbean*	59 (2.1%)	222 (3.4%)	304 (5.0%)	416 (6.5%)	1001 (4.6%)
*– Other*	114 (4.0%)	282 (4.3%)	287 (4.7%)	404 (6.3%)	1087 (5.0%)
*– Unknown*	50 (1.7%)	49 (0.8%)	23 (0.4%)	42 (0.7%)	164 (0.7%)
Acute infection	1319 (46.1%)	3235 (49.8%)	2844 (46.3%)	2624 (40.7%)	10022 (45.7%)
First available CD4 (cells/μl)	524 (350, 740)	490 (348, 670)	500 (360, 663)	503 (370, 676)	501 (359, 678)
Time since SC of first CD4 (months)	19 (5, 78)	5 (2, 9)	4 (2, 6)	3 (2, 5)	4 (2, 7)
Number of CD4 measurements	6 (2, 13)	4 (2, 10)	6 (3, 10)	2 (1, 4)	4 (2, 8)
Time since SC of last CD4 (months)	87 (51, 142)	23 (6, 59)	23 (9, 44)	7 (3, 16)	20 (6, 51)
First available HIV-RNA (log_10_ c/ml)	4.2 (3.5, 4.9)	4.6 (3.9, 5.2)	4.7 (4.0, 5.3)	4.8 (4.2, 5.4)	4.6 (3.9, 5.2)
Time since SC of first HIV-RNA (months)	59 (30, 108)	5 (2, 9)	4 (2, 6)	3 (2, 5)	4 (2, 10)
Number of HIV-RNA measurements	2 (1, 6)	3 (1, 9)	5 (2, 9)	2 (1, 4)	3 (1, 7)
Time since SC of last HIV-RNA (months)	85 (50, 142)	23 (6, 58)	22 (9, 43)	6 (3, 15)	19 (6, 50)

Median and IQR for continuous variables; absolute and relative percentage frequencies for categorical variable.

Average VL was estimated to decrease rapidly during the first year after seroconversion before reaching SPVL, followed by a slow increase thereafter. CD4 was estimated to increase following seroconversion, reaching a peak at approximately 4 months and declining thereafter (Figure S1, Supplemental Digital Content).

Overall, we found evidence of significant trends according to the calendar time of seroconversion in estimated SPVL and peak CD4 (*P* < 0.001 for both). More specifically, SPVL was lowest for those seroconverting in the early 1980s, increasing to reach a plateau for those seroconverting after 2000. We observed opposite trends for peak CD4 with the highest values estimated for those seroconverting in the early 1980s followed by a decline to reach a plateau after the year 2000 approximately. The overall unadjusted estimated (95% CI) average SPVL for those seroconverting in January 1, 1980, January 1, 1995, January 1, 2005, and December 31, 2014 was 3.2 (3.0, 3.4), 4.0 (3.9, 4.1), 4.3 (4.2, 4.4), and 4.2 (4.1, 4.3) log_10_ copies/ml, respectively. The corresponding estimates for peak CD4 was 960 (898, 1025), 615 (584, 648), 521 (492, 551), and 560 (526, 594) cells/μl, respectively.

**Fig. 1:**
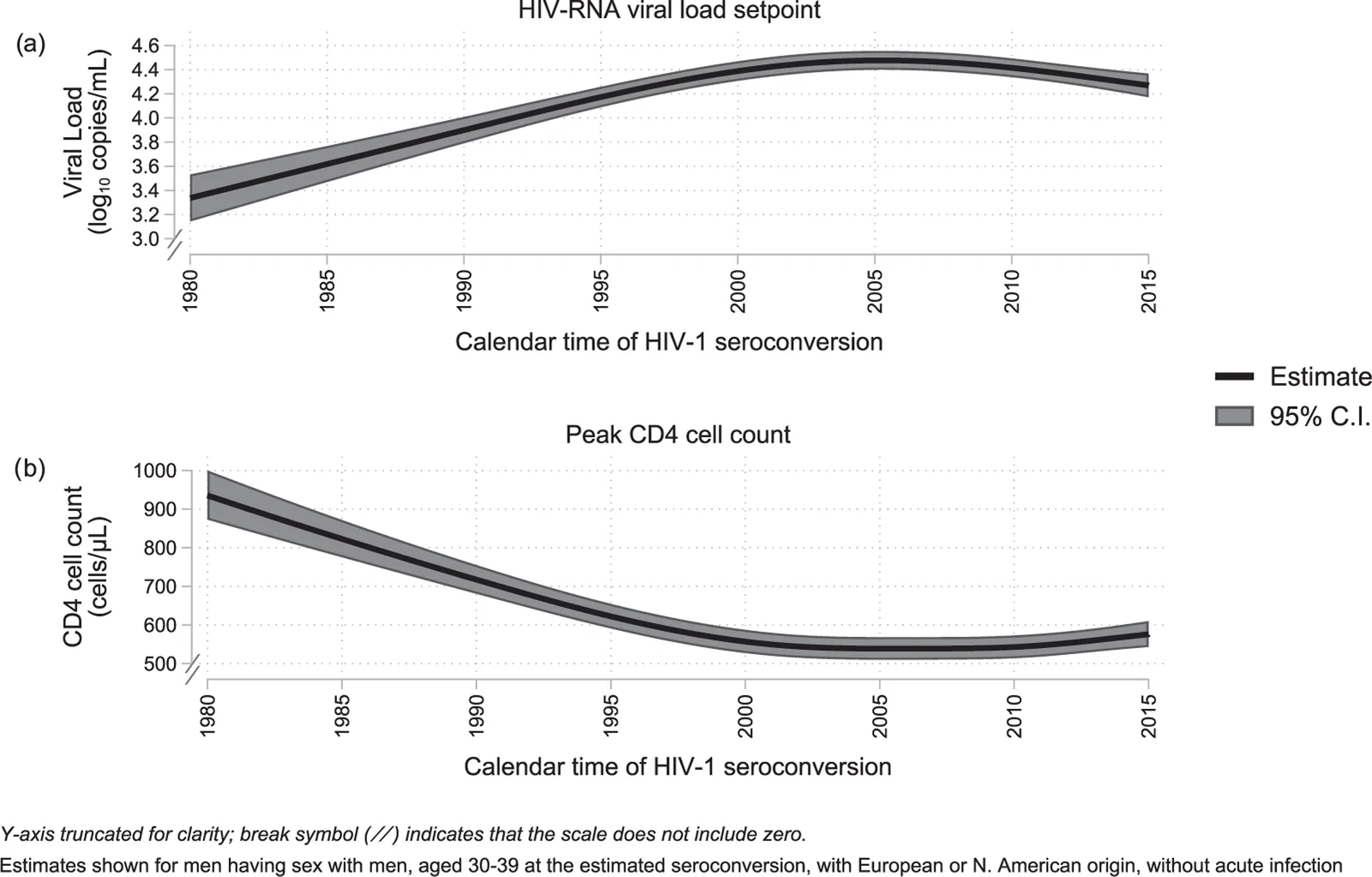
Estimated evolution of (a) HIV-RNA viral load setpoint (at ~1 year after seroconversion) and (b) peak CD4^+^ cell count (at ~4 months after seroconversion) by calendar time of HIV-1 seroconversion.

These trends remained statistically significant (*P* < 0.001 for both SPVL and peak CD4) when parameters were estimated through multivariable mixed models adjusted for sex at birth/ probable route of exposure combinations, age at seroconversion, region of origin, diagnosis during acute infection and VL quantification assay (only for SPVL model). Estimated SPVL and peak CD4 over calendar time of seroconversion for MSM aged 30–39 years at seroconversion of European/North American origin and without acute infection are graphically presented in Fig. 1. It is noteworthy that for both SPVL and peak CD4 the lowest and highest values, respectively, were estimated for those seroconverting in 2005 and the corresponding increasing/decreasing trends show a mild reversal thereafter.

**Fig. 2:**
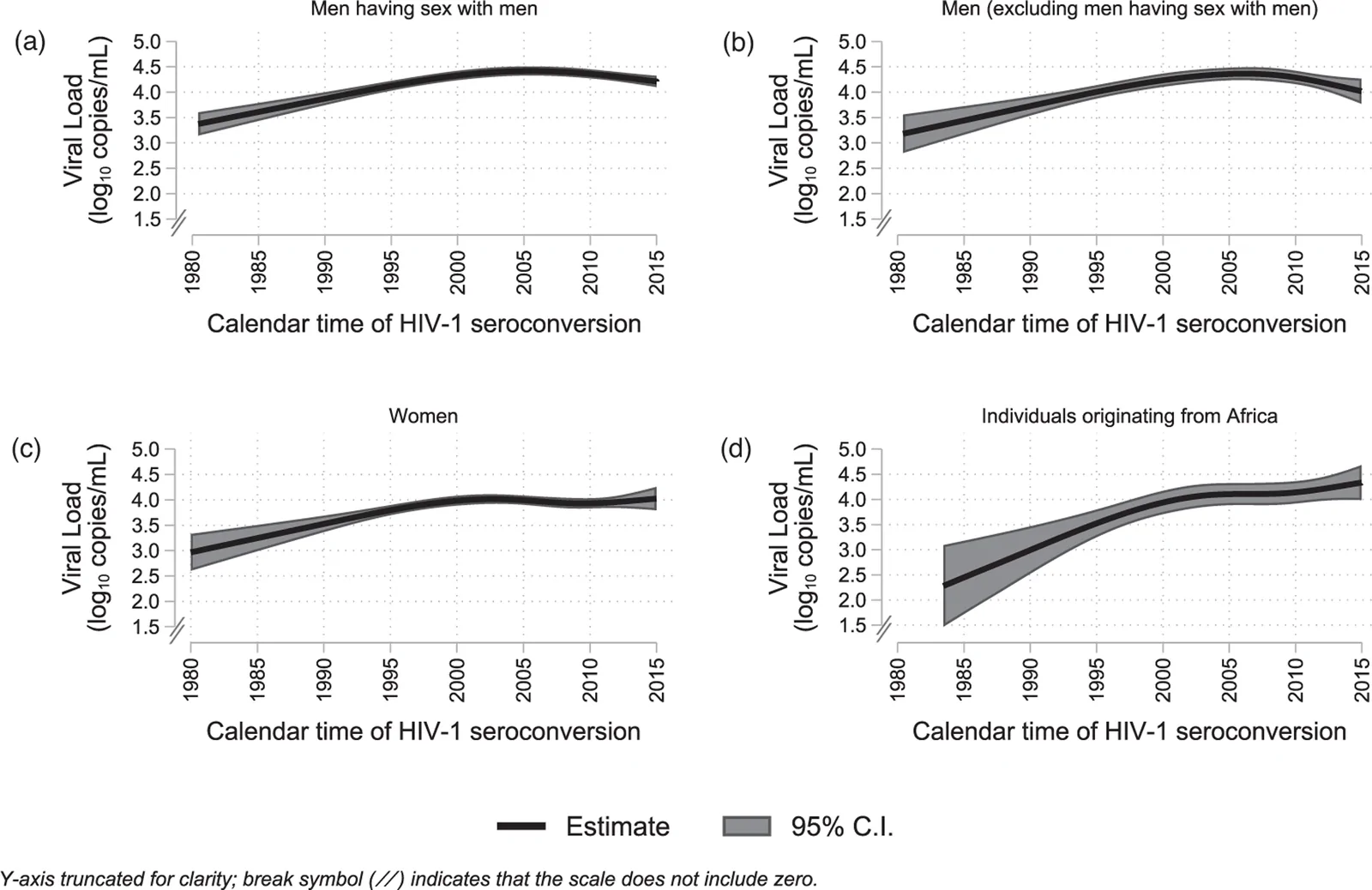
Estimated evolution of HIV-RNA viral load setpoint (at ~1 year after seroconversion) by calendar time of HIV-1 seroconversion in selected subgroups: (a) men having sex with men, (b) men (excluding men having sex with men), (c) women and (d) individuals from Africa.

**Fig. 3:**
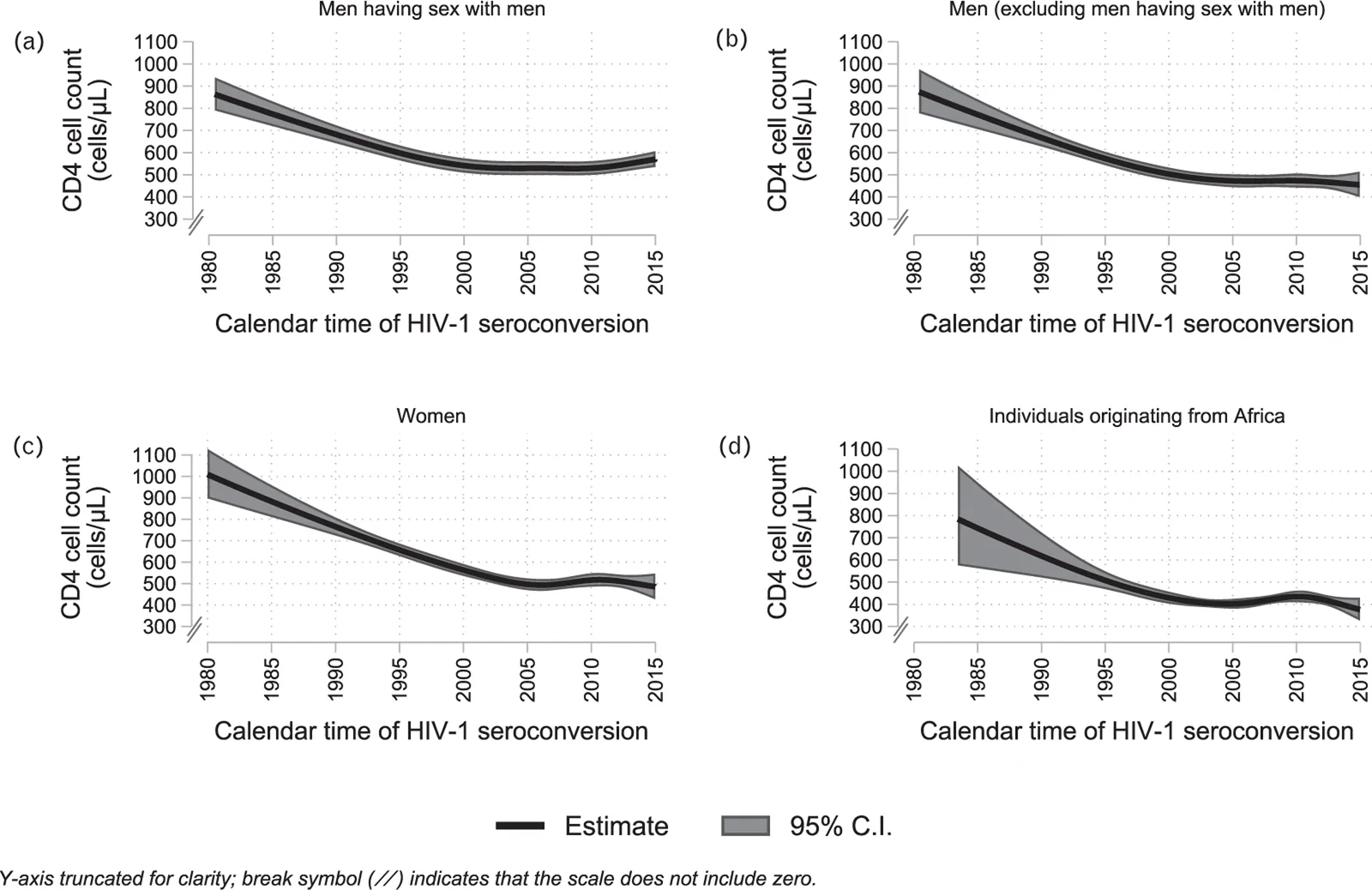
Estimated evolution of peak CD4^+^ cell count (at ~4 months after seroconversion) by calendar time of HIV-1 seroconversion in selected subgroups: (a) men having sex with men, (b) men (excluding men having sex with men), (c) women and (d) individuals from Africa.

Results from unadjusted analyses for specific subgroups of study participants are presented in Fig. 2 for SPVL and Fig. 3 for peak CD4. These results are, in general, consistent with those of the main analysis (Figure 1) but the trend reversal after 2005 seems evident only for MSM for both SPVL (Figure 2a) and peak CD4 (Fig. 3a), and for SPVL for men with other probable routes of exposure (Fig. 2b).

Women having sex with men had lower SPVL (from ~0.2 to ~0.4 log10 copies/ml) and higher peak CD4 (from ~20 to ~80 cells/μl) compared to men having sex with women (irrespective of geographic region of origin) throughout the study period. Men and women originating from Africa had similar SPVL compared to those originating from Europe/North America but lower peak CD4 (~130 to ~180 cells/μl).

Similar analyses in subgroups defined by calendar time of seroconversion and sex at birth/probable route of exposure combinations revealed that changes in the evolution of VL and CD4 during natural history, throughout the epidemic, showed substantial variations across different sex at birth/exposure groups (Figures S3, Supplemental Digital Content and Figure S4, Supplemental Digital Content).

Multivariable models, including interaction terms between calendar time of seroconversion and other covariates (age at seroconversion, sex at birth/exposure groups and region of origin), confirmed that groups defined by sex at birth and probable route of exposure had both different SPVL and peak CD4 as well as different corresponding trends over calendar time (interaction p-value 0.002 for SPVL and 0.008 for peak CD4). Interactions between calendar time of seroconversion and the other examined covariates were not statistically significant. Estimated SPVL and peak CD4 over calendar time of seroconversion for individuals aged 30–39 years at seroconversion of European/North American origin not diagnosed during acute infection for different combinations of sex at birth and probable route of exposure are shown graphically in Figure S2, Supplemental Digital Content

Corresponding estimates for selected combinations of sex at birth, probable route of HIV exposure, region of origin and calendar time of HIV-1 seroconversion are given in Table 2. As shown in this table, there were substantial increases in SPVL of at least 1.0 log_10_ copies/ml from the lowest levels observed in 1980 to the highest observed in 2005 for all subgroups examined. Subsequent trends after 2005 until the end of 2014 were declining but the magnitude of change was much smaller ranging from a drop of 0.2 log_10_ copies/ml for MSM from Europe/North America and men having sex with women from Europe/North America or Africa to a drop of 0.5 log_10_ copies/ml for people who inject drugs from Europe/North America. We observed no evidence of this trend reversal for women having sex with men from either Europe/North America or Africa.

**Table 2:** Estimated (a) viral load setpoint^a^ and (b) peak CD4 cell count^b^ for selected combinations of participant characteristics.

		Calendar time of HIV-1 seroconversion			
Sex at birth and probable route of HIV exposure	Region of origin	January 1, 1980	January 1, 1995	January 1, 2005	December 31, 2014
(a)		Estimated (95% CI) viral load setpoint (log_10_ copies/ml)				
Men sex with men	Europe/N. America	3.4 (3.1, 3.6)	4.1 (4.1, 4.2)	4.4 (4.4, 4.5)	4.2 (4.1, 4.3)
Men sex with women	Europe/N. America	2.9 (2.5, 3.3)	4.0 (3.8, 4.1)	4.4 (4.3, 4.5)	4.2 (4.0, 4.3)
Men sex with women	Africa	2.8 (2.4, 3.2)	3.9 (3.8, 4.0)	4.3 (4.2, 4.4)	4.1 (3.9, 4.3)
Women sex with men	Europe/N. America	2.6 (2.2, 2.9)	3.7 (3.6, 3.8)	4.0 (3.9, 4.1)	4.0 (3.8, 4.1)
Women sex with men	Africa	2.5 (2.2, 2.8)	3.6 (3.5, 3.7)	3.9 (3.8, 4.0)	3.9 (3.7, 4.1)
People who inject drugs	Europe/N. America	3.2 (2.8, 3.5)	4.1 (3.9, 4.2)	4.3 (4.2, 4.4)	3.8 (3.4, 4.2)
(b)		Estimated (95% CI) peak CD4^+^ cell count (cells/μl)			
Men sex with men	Europe/N. America	871 (801, 944)	611 (580, 642)	541 (513, 570)	583 (550, 617)
Men sex with women	Europe/N. America	961 (809, 1126)	597 (560, 635)	511 (477, 546)	542 (486, 602)
Men sex with women	Africa	786 (648, 938)	461 (426, 497)	386 (355, 418)	413 (364, 466)
Women sex with men	Europe/N. America	1038 (920, 1164)	680 (644, 717)	563 (529, 598)	564 (509, 621)
Women sex with men	Africa	856 (747, 973)	535 (500, 570)	431 (401, 463)	432 (384, 483)
People who inject drugs	Europe/N. America	983 (884, 1088)	635 (598, 673)	523 (477, 571)	443 (316, 592)

Estimates are for individuals aged 30–39 years at seroconversion, not diagnosed with acute infection.

a At ~1 year after seroconversion.

b At ~4 months after seroconversion.

Likewise, with peak CD4, all groups had substantial drops from the highest levels observed in 1980 to the lowest in 2005 ranging from a drop of 330 cells/μL for MSM from Europe/North America to a drop of 475 cells/μl for women having sex with men from the same region. The increases in peak CD4 from 2005 to the end of 2014 (trend reversal) were small, ranging from a gain of 1 for women having sex with men from Europe/North America or Africa to a gain of 42 cell/μl for MSM from Europe/North America). For people who inject drugs there was an apparent continuation of a declining trend.

To examine whether the observed temporal trends could be explained by changes in the distribution of HIV-1 subtypes, we repeated the analyses restricted to individuals with subtype B infection. Subtype information was available for 4360 of 21 933 individuals (20%), of whom 3783 (87%) had HIV-1 subtype B, contributing 23 892 viral load and 29 005 CD4 measurements. The estimated trajectories of setpoint viral load and peak CD4 count were highly similar to those obtained in the full dataset (Figure S5, Supplemental Digital Content), indicating that the overall patterns were not driven by changes in subtype composition.

## Discussion

Over the 20-year period 1980–2000, the SPVL rose and peak CD4 shortly following HIV-1 seroconversion decreased before reaching plateaus to 2005. The dramatic changes we observed of 1.1 log_10_ copies/mL rise in SPVL and 439 cell/μl fall in peak CD4 from 1980 to 2005 appear to indicate increasing HIV-1 virulence and subsequent adaptation to the population as a balance is achieved between transmission potential and host survival.

A rise of more virulent strains may appear alarming, but a recent study reported no association between replication capacity and immune recovery after ART [13]. So perhaps from a clinical perspective an increase in average SPVL in the population is not a major concern, although it remains so in terms of public health given the greater likelihood of transmission. In contrast, the observation that peak CD4 shortly after HIV seroconversion has declined over the study period is of considerable concern. Lower initial CD4 levels are associated with reduced time to clinically significant immunosuppression, increasing the risk of opportunistic infections. Furthermore, initiating ART at lower CD4 has been consistently linked to suboptimal immunologic recovery, potentially leading to poorer treatment outcomes. Public health messages should, therefore, continue to reinforce the need to focus both on prevention of transmission and early diagnosis and treatment.

Beyond clinical and public health implications, our findings also have methodological consequences for modelling and surveillance. CD4 at seroconversion are frequently used by modelers to estimate within-country HIV transmission dynamics and to infer HIV incidence using RITA assays, which assume that individuals diagnosed with CD4 <350 cells/μl represent those with long-term rather than recent infections. The observed decline in early postseroconversion CD4 levels from 1980 to 2005 suggests that these assumptions may no longer be valid and that transmission models relying on them require re-evaluation and recalibration to maintain accuracy.

The increase in virulence markers observed from the early 1980s through 2005 spans both the pre-ART and early ART eras. During the first phase of the epidemic, rising SPVL and declining peak CD4 counts may reflect viral adaptation to a largely untreated host population, with selection favoring strains associated with higher transmission potential and possibly more rapid within-host replication. Combination ART was introduced in 1996–1997, generally including drugs from the protease inhibitor and/or nonnucleoside reverse transcriptase inhibitor classes, followed by the development of better-tolerated agents. While the upward trends in virulence markers had already begun before combination ART became available, the expansion of ART coverage could have sustained or amplified this preexisting pattern.

ART can markedly reduce HIV-1 transmission [14], and this reduction in transmission opportunities may favor viruses with higher virulence to maintain transmission potential. Indeed, modelling studies have predicted that mean SPVL will increase as treatment rollout expands [15,16]. Furthermore, levels of transmitted and acquired drug resistance were relatively high during that period [17–19], which may have allowed ongoing transmission from individuals on ART despite partial viral suppression. Although most resistance mutations reduce viral fitness [20], modelling studies have suggested that incomplete suppression at the population level could still contribute to an apparent increase in mean SPVL at the population level [21].

Post 2005, however, these trends appear to have reversed during a period marked by continuing improvement in drug formulation, decreasing levels of drug resistance, and the introduction of drugs from the integrase strand transfer inhibitor class, which remain the recommended first line treatment. On further examination, this reversal appears to be restricted to MSM as well as men who do not report sex with men suggesting decreasing virulence in the circulating strains. A recent phylogenetic study by Wymant *et al.* reported on a cluster of 109 MSM who appear to have acquired a particularly virulent subtype-B variant, which possibly arose in the 1990 s, compared to other subtype-B strains [15]. Our findings indicate that this may be a limited cluster, however, which has not become generalized in MSM populations in Europe and support a modelling study by Stansfield *et al.* examining the potential effect of test-and-treat on HIV-1 virulence among MSM [16]. The study reported that increasing treatment coverage leads to increased virulence, although once MSM behavioral data are considered, increasing treatment coverage selected for lower virulence. It is noteworthy that a similar trend reversal was also observed among men who did not report sex with men, although the reasons for this pattern remain unclear. Investigating potential transmission links between population groups would require behavioral and/or sequence-based data, which were not available in the present analysis.

The lower SPVL and higher peak CD4 and, indeed, overall lower VL and higher CD4 reported here for women compared to their male counterparts supports our previous findings and those of others [17,18]. It has been proposed that this may be due to estrogen inhibiting viral replication [19], as well as latency control [22], and may shed some light on why HIV-1 elite controllers [23], and post treatment controllers [24] are more likely to be women.

We also found that SPVL for individuals of African origin is lower and with lower levels of viremia persisting throughout our study period. We previously reported that Africans, especially those recruited in Europe, had lower CD4 at seroconversion and slower CD4 decline than non-African Europeans [25] and that this may relate to viral subtype, although this information was missing for most participants. Certainly, studies have reported on replication capacity being higher for subtype B, the most common subtype in western Europe and North America, compared to non-B subtypes [13,26]. In previous analysis of 3364 seroconverters with known subtype (86% B) we also found a tendency for all subtypes, except C, to have lower SPVL levels compared with subtype B [27]. The lower peak CD4 counts observed among participants originating from Africa may partly reflect lower baseline CD4 levels in populations without HIV from Sub-Saharan Africa [28] rather than intrinsic differences in HIV-1 virulence or disease progression. Because preinfection CD4 measurements were not available, we cannot disentangle these effects.

There are several limitations worthy of discussion. Firstly, the interval between seroconversion and the first recorded CD4 count was longer and more variable in the early period (1980–1995), with a median of 19 months, and shortened thereafter to 5, 4, and 3 months in later periods, possibly reflecting changes in clinical monitoring and data completeness. Our analyses though modelled longitudinal CD4 trajectories during untreated infection and estimated peak CD4 at approximately 4 months after seroconversion, rather than relying on the timing of the first CD4 measurement. If individuals with more advanced disease were preferentially monitored earlier in the epidemic, this would be expected to lower, rather than raise, early CD4 estimates, suggesting that the observed decline in peak CD4 over calendar time is unlikely to be explained by changes in measurement timing.

Secondly, because HIV-1 subtype information was available only for a subset of individuals, we cannot fully exclude that the observed changes reflect, at least in part, shifts in circulating viral subtypes within specific populations. However, in analyses restricted to individuals with subtype B infection (Figure S5, Supplemental Digital Content), the temporal trends in both setpoint viral load and peak CD4 count closely mirrored those observed in the overall population, suggesting that our main findings are unlikely to be driven by changes in subtype distribution.

Thirdly, we lack reference data on temporal changes in CD4 counts in HIV-negative populations, which may also have changed over time; this limitation prevents us from assessing whether part of the observed trend reflects broader population-level influences unrelated to HIV infection.

Fourthly, viral load assays have become progressively more sensitive over calendar time, with lower limits of quantification and improved precision across the measurement range. However, our analysis adjusted for assay used and the trends observed remained.

Our study has several strengths, however, not least its size making it the largest study of HIV-1 seroconverters. Data included spanned the HIV-1 epidemic in the global west from the 1980 s to the start of test-and-treat era in 2015. And, although data are mostly from MSM, we have sufficient numbers which enabled us to also examine temporal trends within other key subgroups.

In conclusion, our findings of secular changes in SPVL are supported by the contemporaneous changes in peak CD4. Both measures provide important information on the circulating strains and should ideally be monitored overall and in subgroups. Our findings have implications for surveillance and modelling efforts that rely on early CD4 and viral load levels. Since we can no longer examine setpoint viremia due to the early initiation of ART, such monitoring will need to rely on peak CD4 trends and viral fitness assays.

## Acknowledgements

We would like to thank all people living with HIV-1 who agreed to participate in the CASCADE cohorts and on whose altruism we depend for our research.

We thank ViiV Healthcare UK, Janssen Pharmaceutica NV and Merck Sharp & Dohme for funding the study.

We also acknowledge the funding bodies, executive and steering committees of the constituent CASCADE cohorts, and are immensely grateful to colleagues in clinical centers providing HIV care and recruiting study participants. We are also grateful to Dr Alvaro Passi Solar for the setup and management of the study database and to Mrs Phoebe Day-Lloyd for administrative support.

This work was presented in part at the Conference on Retroviruses and Opportunistic Infections, Denver, USA, 3–6 March 2024 [abstract number 01044].

Author contributions: All authors had final responsibility for the decision to submit for publication. F.B. and N.P. had full access to all the data in the study. Conceptualization: N.P. Data curation: D.C., A.v.S., I.J., L.M., C.S., C.C., J.G., S.T., B.S., F.B., G.T., S.G. Formal analysis: N.P. Funding acquisition: F.B. Methodology: N.P. Supervision: G.T. Writing the original draft: N.P. and G.T. Writing – reviewing and editing: all authors contributed equally. N.P. and F.B. have directly accessed and verified the underlying data reported in the manuscript

Data sharing: Data collected for the study can be shared under a signed data access agreement with bona fide researchers and only after approval of a proposal by CASCADE's executive and scientific committees.

### Conflicts of interest

Dominique Costagliola reports personal fees from Pfizer (2022) for a lecture outside the submitted work. John Gill reports advisory board fees from Merck, Gilead and ViiV. Nikos Pantazis has received grants unrelated to this study and paid to his institution from Gilead Sciences Hellas and ECDC. Fiona Burns reports an institutional grant and personal fees from Gilead Sciences Ltd. Christina Carlander has received lecture, moderator, and advisory board fees from GSK/ViiV, Gilead Sciences and MSD paid to her institution and unrestricted Nordic Fellowship grants from Gilead Sciences Nordic unrelated to this study. Giota Touloumi has received grants unrelated to this study and paid to her institution from Gilead Sciences, UCL UK, EU structural funds and National Funds. The rest of the authors have no competing interests to declare.

Funding/support: This research is funded by ViiV Healthcare UK, Janssen Pharmaceutica NV and Merck Sharp & Dohme.

For CASCADE Collaboration: see Appendix, Supplemental Digital Content.

## Supplementary Material

Supplemental Digital Content

## References

[1] Coffin JM. HIV population dynamics in vivo: implications for genetic variation, pathogenesis, and therapy. *Science* 1995; 267:483–489. 7824947 10.1126/science.7824947

[2] Fraser C Hollingsworth TD Chapman R de Wolf F Hanage WP. Variation in HIV-1 set-point viral load: epidemiological analysis and an evolutionary hypothesis. *Proc Natl Acad Sci USA* 2007; 104:17441–17446. 17954909 10.1073/pnas.0708559104 PMC2077275

[3] Anderson RM May RM. Coevolution of hosts and parasites. *Parasitology* 1982; 85 (Pt 2):411–426. 6755367 10.1017/s0031182000055360

[4] Plana M García F Gallart T Tortajada C Soriano A Palou E. Immunological benefits of antiretroviral therapy in very early stages of asymptomatic chronic HIV-1 infection. *AIDS* 2000; 14:1921–1933. 10997396 10.1097/00002030-200009080-00007

[5] Zanni MV Toribio M Robbins GK Burdo TH Lu MT Ishai AE. Effects of antiretroviral therapy on immune function and arterial inflammation in treatment-naive patients with human immunodeficiency virus infection. *JAMA Cardiol* 2016; 1:474. 27438325 10.1001/jamacardio.2016.0846 PMC5944348

[6] Antiretroviral Therapy Cohort Collaboration. Life expectancy of individuals on combination antiretroviral therapy in high-income countries: a collaborative analysis of 14 cohort studies. *Lancet* 2008; 372:293–299. 18657708 10.1016/S0140-6736(08)61113-7 PMC3130543

[7] Mellors JW Muñoz A Giorgi JV Margolick JB Tassoni CJ Gupta P. Plasma viral load and CD4^+^ lymphocytes as prognostic markers of HIV-1 infection. *Ann Intern Med* 1997; 126:946–954. 9182471 10.7326/0003-4819-126-12-199706150-00003

[8] Pantazis N Porter K Costagliola D De Luca A Ghosn J Guiguet M. Temporal trends in prognostic markers of HIV-1 virulence and transmissibility: an observational cohort study. *Lancet HIV* 2014; 1:e119–126. 26424120 10.1016/S2352-3018(14)00002-2

[9] Rios A. Fundamental challenges to the development of a preventive HIV vaccine. *Curr Opin Virol* 2018; 29:26–32. 29549802 10.1016/j.coviro.2018.02.004

[10] Rambaut A Posada D Crandall KA Holmes EC. The causes and consequences of HIV evolution. *Nat Rev Genet* 2004; 5:52–61. 14708016 10.1038/nrg1246

[11] Ruiz-Burga E Tariq S Touloumi G Gill J Nicholls EJ Sabin C. CASCADE protocol: exploring current viral and host characteristics, measuring clinical and patient-reported outcomes, and understanding the lived experiences and needs of individuals with recently acquired HIV infection through a multicentre mixed-methods observational study in Europe and Canada. *BMJ Open* 2023; 13:e070837. 10.1136/bmjopen-2022-070837 PMC10186449 37169505

[12] Harrell FE, Regression J. Modeling strategies: with applications to linear models, logistic regression, and survival analysis. New York: Springer; 2001.

[13] Rindler AE Kusejko K Kuster H Neumann K Leemann C Zeeb M. The interplay between replication capacity of HIV-1 and surrogate markers of disease. *J Infect Dis* 2022; 226:1057–1068. 35299248 10.1093/infdis/jiac100

[14] Rodger AJ Cambiano V Bruun T Vernazza P Collins S van Lunzen J. Sexual activity without condoms and risk of HIV transmission in serodifferent couples when the HIV-positive partner is using suppressive antiretroviral therapy. *JAMA* 2016; 316:171–181. 27404185 10.1001/jama.2016.5148

[15] Wymant C Bezemer D Blanquart F Ferretti L Gall A Hall M. A highly virulent variant of HIV-1 circulating in the Netherlands. *Science* 2022; 375:1979. 10.1126/science.abk1688 35113714

[16] Stansfield SE Herbeck JT Gottlieb GS Abernethy NF Murphy JT Mittler JE. Test-and-treat coverage and HIV virulence evolution among men who have sex with men. *Virus Evol* 2021; 7:veab011. 33633867 10.1093/ve/veab011 PMC7893213

[17] Touloumi G Pantazis N Babiker AG Walker SA Katsarou O Karafoulidou A. Differences in HIV RNA levels before the initiation of antiretroviral therapy among 1864 individuals with known HIV-1 seroconversion dates. *AIDS* 2004; 18:1697–1705. 15280781 10.1097/01.aids.0000131395.14339.f5

[18] Sterling TR Vlahov D Astemborski J Hoover DR Margolick JB Quinn TC. Initial plasma HIV-1 RNA levels and progression to AIDS in women and men. *N Engl J Med* 2001; 344:720–725. 11236775 10.1056/NEJM200103083441003

[19] Szotek EL Narasipura SD Al-Harthi L. 17β-Estradiol inhibits HIV-1 by inducing a complex formation between β-catenin and estrogen receptor α on the HIV promoter to suppress HIV transcription. *Virology* 2013; 443:375–383. 23769242 10.1016/j.virol.2013.05.027 PMC3722310

[20] Martinez-Picado J Martínez MA. HIV-1 reverse transcriptase inhibitor resistance mutations and fitness: a view from the clinic and ex vivo. *Virus Res* 2008; 134:104–123. 18289713 10.1016/j.virusres.2007.12.021

[21] Herbeck JT Mittler JE Gottlieb GS Goodreau SM Murphy JT Cori A. Evolution of HIV virulence in response to widespread scale up of antiretroviral therapy: a modeling study. *Virus Evol* 2016; 2:vew028. 29492277 10.1093/ve/vew028 PMC5822883

[22] Das B Dobrowolski C Luttge B Valadkhan S Chomont N Johnston R. Estrogen receptor-1 is a key regulator of HIV-1 latency that imparts gender-specific restrictions on the latent reservoir. *Proc Natl Acad Sci USA* 2018; 115:E7795–E7804. 30061382 10.1073/pnas.1803468115 PMC6099847

[23] de Azevedo SSD Caetano DG Côrtes FH Teixeira SLM dos Santos Silva K Hoagland B. Highly divergent patterns of genetic diversity and evolution in proviral quasispecies from HIV controllers. *Retrovirology* 2017; 14:29. 28464889 10.1186/s12977-017-0354-5 PMC5414336

[24] Stöhr W Fidler S McClure M Weber J Cooper D Ramjee G. Duration of HIV-1 viral suppression on cessation of antiretroviral therapy in primary infection correlates with time on therapy. *PLoS One* 2013; 8:e78287. 24205183 10.1371/journal.pone.0078287 PMC3808338

[25] Pantazis N Morrison C Amornkul PN Lewden C Salata RA Minga A. Differences in HIV natural history among African and non-African seroconverters in Europe and seroconverters in sub-Saharan Africa. *PLoS One* 2012; 7:e32369. 22412867 10.1371/journal.pone.0032369 PMC3295758

[26] Ariën KK Abraha A Quiñones-Mateu ME Kestens L Vanham G Arts EJ. The replicative fitness of primary human immunodeficiency virus type 1 (HIV-1) group M, HIV-1 group O, and HIV-2 isolates. *J Virol* 2005; 79:8979–8990. 15994792 10.1128/JVI.79.14.8979-8990.2005 PMC1168791

[27] Touloumi G Pantazis N Pillay D Paraskevis D Chaix ML Bucher HC. Impact of HIV-1 subtype on CD4 count at HIV seroconversion, rate of decline, and viral load set point in European seroconverter cohorts. *Clin Infect Dis* 2013; 56:888–897. 23223594 10.1093/cid/cis1000

[28] Malaza A Mossong J Bärnighausen T Viljoen J Newell ML. Population-based CD4 counts in a rural area in South Africa with high HIV prevalence and high antiretroviral treatment coverage. *PLoS One* 2013; 8:e70126. 23894603 10.1371/journal.pone.0070126 PMC3720940

