# Supplementary material for: Temporal trends in viral setpoint and peak CD4+ cell count soon after HIV-1 seroconversion
Source: AIDS. 2026 Apr 22;40(9):1346–54. doi: 10.1097/QAD.0000000000004527 (PMC13566474; doi:10.1097/QAD.0000000000004527)
Supplement: Supplementary file 1 [file aids-40-1346-s001.docx]

**Appendix**

**CASCADE Collaboration**

CASCADE Scientific Steering Committee: John Gill (co-chair), Kholoud Porter (co-chair), Christina Carlander, Rafael Eduardo Campo, Harmony Garges, Sophie Grabar, Inma Jarrín, Laurence Meyer, Moustafa Kamel, Giota Touloumi, Marc van der Valk, Vani Vannappagari, Alain Volny Anne, Linda Wittkop, Lital Young.

The ANRS PRIMO Cohort is supported by ANRS|MIE. The Scientific Committee comprises: Sophie Abgrall, Valerie Andriantsoanirina, Veronique Avettand-Fenoel, Christine Bourgeois, Marie-Laure Chaix, Antoine Cheret, Hugues Fischer, Cecile Goujard, Caroline Lascoux-Combe, Annie Le Palec, Laurence Meyer, Ventzlislava Petrov-Sanchez, Asier Saez-Cirion, Remonie Seng, Bruno Spire, Karl Stefic, Josephine Tine.

Financial support for FHDH is through ANRS-MIE, Sorbonne Université, Inserm and the French Ministry of Health. ANRS CO4 FHDH Scientific Committee : E Piet (COREVIH Auvergne Loire); A Gagneux-Brunon, C Jacomet (COREVIH Auvergne Loire); L Piroth (COREVIH Bourgogne Franche-Comté); F Benezit, M Goussef, P Tattevin (COREVIH Bretagne); F Bani Sadr (COREVIH Grand Est); I Lamaury (COREVIH Guadeloupe); M Nacher (COREVIH Guyane); H Bazus, O Robineau (COREVIH Hauts-de-France); R Calin, C Katlama, J L Meynard (COREVIH Île de France Centre); B Denis (COREVIH Île de France Est); J Ghosn, V Joly, M A Khuong (COREVIH Île de France Nord); F Caby, E Rouveix Nordon, P de Truchis (COREVIH Île de France Ouest), S Abgrall, A Chéret, C Duvivier (COREVIH Île de France Sud), A Becker, P Miailhes (COREVIH Lyon Vallée du Rhône), S Abel (COREVIH Martinique), G Unal (COREVIH Normandie); A Makinson, G Martin-Blondel (COREVIH Occitanie); A Morisot (COREVIH PACA-Est); S Bregigeon, P Enel (COREVIH PACA-Ouest); C Allavena, V Rabier (COREVIH Pays-de-la-Loire); H Fischer, L Vallet (Association of PWH : TRT-5 CHV); L Marchand/T Saïdi (ANRS-MIE); D Costagliola and S Grabar (Sorbonne Université, INSERM IPLESP). ANRS CO4 FHDH Steering Commitee: Pr Sophie Abgrall (Béclère), Pr Pierre Tattevin (Rennes), Dr Pierre de Truchis (Garches), M Hughes Fischer (TRT-5 CHV association) and for Inserm Iplesp U1136 Dominique Costagliola (coPI), Dr Sophie Grabar (Principal Investigator).

The CoRIS cohort is supported by CIBER -Consorcio Centro de Investigación Biomédica en Red- (CB21/13/00091), Instituto de Salud Carlos III, Ministerio de Ciencia e Innovación and Unión Europea – NextGenerationEU. CoRIS Executive committee: Santiago Moreno, Inma Jarrín, David Dalmau, M Luisa Navarro, M Isabel González, Federico Garcia, Eva Poveda, Jose Antonio Iribarren, Félix Gutiérrez, Rafael Rubio, Francesc Vidal, Juan Berenguer, Juan González, M Ángeles Muñoz-Fernández.

AMACS: Steering Committee: Adamis G., Chini M., Chrysos G., Marangos M., Kofteridis D., Metallidis S., Panagopoulos P., Pantazis N., Papadopoulos A., Vasalou V., Papastamopoulos V., Psychogiou M., Sambatakou H. (Co-Chair), Sipsas NV., Touloumi G. (Chair). Coordinating and Statistical Analysis Center: Department of Hygiene, Epidemiology and Medical Statistics, Medical School, National and Kapodistrian University of Athens, Greece (Touloumi G., Pantazis N., Thomadakis C.).

The ATHENA database is maintained by Stichting HIV Monitoring and supported by a grant from the Dutch Ministry of Health, Welfare and Sport through the Centre for Infectious Disease Control of the National Institute for Public Health and the Environment.

The Swedish HIV Cohort InfCareHIV is managed by the InfCareHIV Steering Committee and funded by The Swedish Association of Local Authorities and Regions.

UK CASCADE:

UK CASCADE: Julie Fox, Louise Terry (Guy’s and St Thomas Foundation Trust, Harrison wing); Alison Uriel, Pamela Hackney (Manchester University NHS Foundation Trust, North Manchester General Hospital); Sarah Fidler, Wilbert Ayap (Imperial College Healthcare NHS Trust, St Mary’s Hospital); Laura Waters, Fowsiya Nur (Central and Northwest London NHS Foundation Trust); Fiona Burns, Sabine Kinloch, Thomas Fernandez (Royal Free London NHS Foundation Trust); Diarmuid Nugent, Javier Pinedo (56 Dean Street Clinic, Chelsea and Westminster Hospital NHS Foundation Trust); Iain Reeves, Lidia Ignatov, Tracy Fong (Homerton University Hospital Foundation Trust); Laura Cunningham, Jaydee Pangan (Cardiff Royal Infirmary); Claire Mackintosh, Louise Sharp (Western General Hospital, NHS Lothian).

CASCADE cohorts providing data from previous publication

Badalona IDU hospital cohort (Roberto Muga, Jordi Tor), Barcelona IDU Cohort (Patricia Garcia de Olalla, Joan Cayla), Madrid cohort (Julia Del Amo, Jorge del Romero), Valencia IDU cohort (Santiago Pérez-Hoyos), Italian Seroconversion Study (Giovanni Rezza, Maria Dorrucci), ANRS CO2 SEROCO cohort (Laurence Meyer, Faroudy Boufassa), German HIV-1 seroconverter cohort (Barbara Gunsenheimer-Bartmeyer, Uwe Koppe, Karolin Meixenberger).

***Ethics committees***

The following ethics committees revised and approved the protocols to pool clinical data following informed signed consent from study participants: AMACS cohort (Greece): Bioethics and Deontology Committee of the Medical School of the National and Kapodistrian University of Athens and National Organisation of Medicines; ANRS CO4 French Hospital database on HIV (France): Commission Nationale de l’informatique et des Libertés; ATHENA cohort (Netherlands): Institutional review board; Badalona IDU Hospital Cohort (Spain): Comité Ético de Investigación Clínica del Hospital Universitari Germans Trias i Pujol; CoRIS cohort (Spain): Research Ethics Committee H. General Universitario Gregorio Marañon and the Research Ethics Committee of the Institute of Health Carlos III; German HIV-1 Seroconverter Study (Germany): Charité, University Medicine Berlin; Italian Seroconversion Study (Italy) : Comitato etico dell’Istituto Superiore di Sanità; Madrid Cohort (Spain): Ethics Committee of Universidad Miguel Hernandez de Elche; PRIMO cohort (France): Avis initial du CPP; SEROCO Cohort (France): Commission Nationale de l'Informatique et des Libertés (CNIL); Southern Alberta cohort (Canada) : Conjoint Health Research Ethics Board (CHREB), University of Calgary; Swedish cohort (Sweden): Ethics Review Authority; UK Register of HIV Seroconverters (UK): Yorkshire and the Humber South Yorkshire Research Ethics Committee; Valencia IDU Cohort (Spain): Comité Etico de Investigación Clínica del Hospital Dr. Peset-Valencia
