# Supplementary material for: Temporal trends in viral setpoint and peak CD4+ cell count soon after HIV-1 seroconversion
Source: AIDS. 2026 Apr 22;40(9):1346–54. doi: 10.1097/QAD.0000000000004527 (PMC13566474; doi:10.1097/QAD.0000000000004527)
Supplement: Supplementary file 2 [file aids-40-1346-s002.docx]

**Supplementary materials**

**Figure S1**. Estimated evolution of a) HIV-RNA viral load and b) CD4 cell count by time since HIV-1 seroconversion.

*Peak CD4 cell count levels reached at approximately 4 months and setpoint HIV-RNA viral load at approximately 1 year after seroconversion.*

**Figure S2**. Estimated evolution of a) HIV-RNA viral load setpoint (at ~1 year after seroconversion) and b) peak CD4 cell count (at ~4 months after seroconversion) by calendar time of HIV-1 seroconversion.

**Figure S3**. Evolution of HIV-RNA viral load by time since HIV-1 seroconversion (SC) in subgroups of individuals who seroconverted in different calendar periods and with different combinations of sex at birth with probable routes of HIV acquisition.

**Figure S4**. Evolution of CD4 cell count by time since HIV-1 seroconversion (SC) in subgroups of individuals who seroconverted in different calendar periods and with different combinations of sex at birth with probable routes of HIV acquisition.

**Figure S5.** Estimated evolution of a) HIV-RNA viral load setpoint (at ~1 year after seroconversion) and b) peak CD4 cell count (at ~4 months after seroconversion) by calendar time of HIV-1 seroconversion, restricted to individuals with subtype B infection.
